# Ultrastructural Analysis of Large Japanese Field Mouse (*Apodemus speciosus*) Testes Exposed to Low-Dose-Rate (LDR) Radiation after the Fukushima Nuclear Power Plant Accident

**DOI:** 10.3390/biology13040239

**Published:** 2024-04-04

**Authors:** Marta Gatti, Manuel Belli, Mariacarla De Rubeis, Syun Tokita, Hikari Ikema, Hideaki Yamashiro, Yohei Fujishima, Donovan Anderson, Valerie Swee Ting Goh, Hisashi Shinoda, Akifumi Nakata, Manabu Fukumoto, Tomisato Miura, Stefania Annarita Nottola, Guido Macchiarelli, Maria Grazia Palmerini

**Affiliations:** 1 Department of Anatomy, Histology, Forensic Medicine and Orthopaedics, Sapienza University, 00161 Rome, Italy; marta.gatti@uniroma1.it (M.G.); mariacarla.derubeis@uniroma1.it (M.D.R.);; 2 Department of Human Sciences and Promotion of the Quality of Life, San Raffaele Roma Open University, 00166 Rome, Italy; manuel.belli@uniroma5.it; 3 Graduate School of Science and Technology, Niigata University, Niigata 959-2181, Japan; 4 Department of Risk Analysis and Biodosimetry, Institute of Radiation Emergency Medicine, Hirosaki University, Aomori 036-8564, Japanande4163@hirosaki-u.ac.jp (D.A.); tomisato@hirosaki-u.ac.jp (T.M.); 5 Department of Radiobiology, Singapore Nuclear Research and Safety Initiative, National University of Singapore, Singapore 138602, Singapore; 6 Graduate School of Dentistry, Tohoku University, Sendai 980-8575, Japan; 7 Department of Life Science, Faculty of Pharmaceutical Sciences, Hokkaido University of Science, Hokkaido 006-8585, Japan; 8 RIKEN Center for Advanced Intelligence Project, Pathology Informatics Team, Tokyo 103-0027, Japan; manabu.fukumoto.a8@tohoku.ac.jp; 9 Department of Life, Health and Environmental Sciences, University of L’Aquila, 67100 L’Aquila, Italy; guido.macchiarelli@univaq.it

**Keywords:** Fukushima accident, ionizing radiation, ultrastructure, testis, environmental pollution, spermatogenesis

## Abstract

**Simple Summary:**

In 2011, the Fukushima Daiichi Nuclear Power Plant (FDNPP) accident resulted in the release of vast quantities of radioactive substances. In this study, we performed an ultrastructural analysis of the testis of the wild large Japanese field mouse (*Apodemus speciosus*), inhabiting the area near the ex-evacuation zones of the FDNPP and chronically exposed to various levels of low-dose-rate (LDR) radiation, to evaluate the effects of ionizing radiation on the male reproductive system and the possible consequences for future generations. Our results showed a preserved morphology of seminiferous tubules. However, ultrastructural changes, such as wide intercellular spaces, cytoplasmic vacuolization, vacuolated mitochondria, and increased lipid droplet clusters, were found, which could be linked to the mechanisms related to spermatogenesis. Long-term chronic LDR radiation exposure associated with the FDNPP accident had no pronounced adverse effect on spermatogenesis in *A. speciosus*, even if testis presented some ultrastructural changes. Our findings could be used in future studies to evaluate the effects of radiation on reproductive health in wild animals.

**Abstract:**

Since the Fukushima Daiichi Nuclear Power Plant (FDNPP) accident, great attention has been paid to the impact of chronic low-dose-rate (LDR) radiation exposure on biological systems. The reproductive system is sensitive to radiation, with implications connected to infertility. We investigated the testis ultrastructure of the wild large Japanese field mouse (*Apodemus speciosus*) from three areas contaminated after the FDNPP accident, with different levels of LDR radiation (0.29 µSv/h, 5.11 µSv/h, and 11.80 µSv/h). Results showed good preservation of the seminiferous tubules, comparable to the unexposed animals (controls), except for some ultrastructural modifications. Increases in the numerical density of lipid droplet clusters in spermatogenic cells were found at high levels of LDR radiation, indicating an antioxidant activity rising due to radiation recovery. In all groups, wide intercellular spaces were found between spermatogenic cells, and cytoplasmic vacuolization increased at intermediate and high levels and vacuolated mitochondria at the high-level. However, these findings were also related to the physiological dynamics of spermatogenesis. In conclusion, the testes of *A. speciosus* exposed to LDR radiation associated with the FDNPP accident showed a normal spermatogenesis, with some ultrastructural changes. These outcomes may add information on the reproductive potential of mammals chronically exposed to LDR radiation.

## 1. Introduction

Following the Fukushima Daiichi Nuclear Power Plant (FDNPP) accident in 2011, substantial amounts of radionuclides were released into the environment, including radioactive iodine, caesium, tellurium, and inert gases [1,2]. Despite it being more than a decade since the accident, the potential long-term effects of exposure to radioactive substances on biological systems continue to be a major concern. For non-human biota, the United Nations Scientific Committee on the Effects of Atomic Radiation (UNSCEAR) suggested the effects of ionizing radiation at the population level could be observed above the chronic dose rate of 100 μGy/h [3]. However, several studies have reported negative effects at lower chronic dose rates on wild animals living in the ex-evacuation zone. These studies have documented morphological alterations in the pale grass blue butterfly (*Pseudozizeeria maha*) [4], a reduction in the number of common birds [5], a decrease in reproductive activity in the goshawk (*Accipiter gentilis*) [6], and changes in proteomic biomarker expression in wild field mice [7]. Nevertheless, the recent analysis demonstrated no significant adverse effects in wildlife chronically exposed to low-dose-rate (LDR) radiation [8,9,10,11]. Such studies emphasize the importance of continued monitoring and mitigation efforts to protect both wildlife and ecosystems from the lingering impacts of radiation exposure.

The large Japanese field mouse (*Apodemus speciosus*) is a suitable species for studying the effects of environmental stress and ionizing radiation. This small mammal is common in Japan, it shows seasonal changes in seminiferous tubule morphology due to its breeding season and serves as a reference animal for the surrounding ecosystem [12,13]. Its reproductive function can be affected by physical and chemical changes as the testis is one of the most known radiosensitive organs, consisting of germ cells at different developmental stages of the spermatogenic cycle [14,15,16,17,18]. The susceptibility to radiation exposure differs between spermatogonia and spermatocytes, according to spermatogenic stages [19,20]. Spermatogenesis during the seasonal reproductive cycle is controlled by mechanisms of proliferation and apoptosis of germ cells [21], which could be sensitive to ionizing radiation because of the high turnover. In light of these considerations, spermatogenesis represents a valuable indicator of ecological and anthropogenic factors affecting animal reproduction.

Studies on LDR (<6 mGy/h) radiation are characterized by limited data and sparse research, leading to significant uncertainties. These uncertainties have contributed to discrepancies in reported effects and their attribution to specific radiation doses [22,23]. Research examining the effects of chronic exposure to LDR radiation on reproductive functions in mammals, including the large Japanese field mouse, has yielded conflicting results [24,25]. Particularly, Takino and colleagues [19] (2017) investigated the effect of chronic LDR radiation on spermatogenic cells in *A. speciosus*, finding enhanced spermatogenesis. In contrast, significant changes were not observed in raccoon testes after long-term exposure to ionizing radiation [26], or in the in vitro fertilizing capacity of cryopreserved sperm of *A. speciosus* after chronic LDR radiation exposure [27].

Nevertheless, the multidisciplinary data are still few and stirring controversy on the impact of ionizing radiation on male fertility in mammals. Transmission electron microscopy (TEM) serves as an invaluable research instrument, enabling the observation of cellular ultrastructure at both organelle and sub-organelle levels. It offers insights into physiological and pathological phenomena that might remain unnoticed using alternative methods.

To our knowledge, the available clinical outcomes and biomolecular data are still poorly sustained by a morphological evaluation of the male reproductive system exposed to ionizing radiation.

Given the limited number of studies on this topic, further research is warranted to comprehensively investigate the effects of LDR radiation on reproductive functions in the large Japanese field mouse. Additionally, the International Commission on Radiological Protection (ICRP) employs a hypothetical model called the “Reference Rat”, representing a standardized mammalian organism used for radiological protection purposes [28]. Studies conducted on this hypothetical rat, alongside research on other animals, are instrumental in establishing dose limits, dose coefficients, and other radiological protection quantities applicable to humans. By using the Reference Rat and examining its responses to radiation exposure, scientists gain valuable insights that aid in understanding the potential risks and impacts of ionizing radiation on reproductive systems and overall radiological risk assessment. The ICRP’s framework of “Reference Animals and Plants”, which includes the Reference Rat, is designed for evaluating the effects of radiation on non-human organisms. This model, applicable to rodents within the Muridae family, including *A. speciosus*, serves as a valuable tool for addressing the diverse scenarios in which these animals may encounter ionizing radiation in their natural environment. Our research on these reference organisms aims to provide valuable insights into assessing the probability and severity of the likely effects of radiation exposure on such individuals. This work not only enhances our understanding of radiation’s impact on wildlife but also contributes to the development of strategies to mitigate potential harm to ecosystems and the environment. 

In this present study, we aimed to evaluate the testicular ultrastructure in *A. speciosus* exposed to low, intermediate, and high levels of LDR radiation as a result of the FDNPP accident and to assess their possible impact on male reproductive functions in wild animals by providing morphological findings.

## 2. Materials and Methods

### 2.1. Ethics

The principles of laboratory animal care were followed during this study, and all procedures were conducted under the guidelines provided by the Ethics Committee for Care and Use of Laboratory Animals for Research of Niigata University, Japan (Protocol No. 26-80-2). The capture of wild rodents in the sampling area was permitted by the Niigata City Office.

### 2.2. Collection of Animals

Large Japanese field mice (*A. speciosus*) were captured in April 2018, during the breeding season, at three contaminated sites, with varying levels of LDR radiation: Tanashio area (low levels of LDR radiation, median value of ambient dose rate: 0.29 μSv/h; range dose rate: 0.21–0.35 µSv/h), Ide area (intermediate levels of LDR radiation, median value of ambient dose rate: 5.11 μSv/h; range dose rate: 4.83–5.77 µSv/h), and Omaru area (high levels of LDR radiation, median value of ambient dose rate: 11.80 μSv/h; range dose rate:10.40–13.10 µSv/h). Contaminated sampling areas were in the Fukushima Prefecture and located within a 20 km radius from the ex-evacuation zone of the FDNPP. 

**Figure 1:**
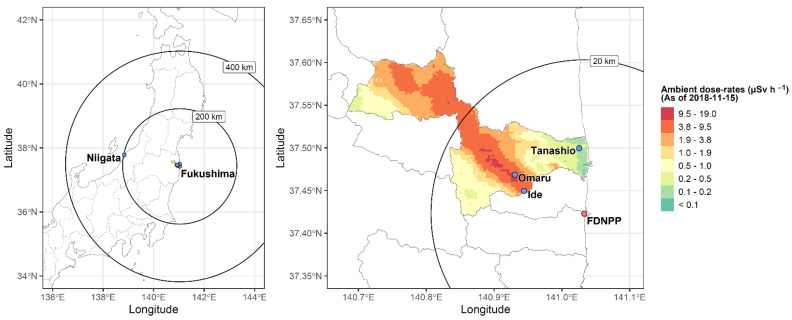
Sampling areas of *A. speciosus* in Fukushima and Niigata Prefectures.

Mice were also collected from an uncontaminated site in Niigata Prefecture (Kakuta area) and used as a control group (Figure 1). Individual data for each large Japanese field mouse are summarized in Table 1. 

Wild mice were captured using Sherman-type live traps baited with peanuts, as previously described [27]. Subsequently, the mice were sacrificed via cervical dislocation on the day of capture. The body weight was measured after the animals were euthanized. It was difficult to determine the age of the mice; therefore, large Japanese field mice with a body weight of more than 17 g were included in the present study. The testes were isolated from each mouse and immediately fixed for ultrastructural analysis in 2.5% glutaraldehyde (Sigma-Aldrich, St. Louis, MO, USA) in phosphate-buffered saline (PBS).

The detailed map represents the control area in Niigata and contaminated areas in Namie Town, Fukushima (Omaru, Ide, Tanashio). The maps were modified shapefiles from the National Land Numerical Information download service, Japan (data were retrieved from http://nlftp.mlit.go.jp/ksj/index.html on 5 February 2016). The heat map shows the ambient dose rates on 15 November 2018 from the 13th airborne monitoring survey conducted by the Nuclear Regulation Authority, Japan (data were retrieved from https://radioactivity.nra.go.jp/ja/list/362/list-1.html on 10 October 2023).

### 2.3. Ambient Radiation Dose rate Measurements at Sampling Sites

The ambient dose rates at each sampling site were measured 1 m above the ground with a NaI (Tl) scintillation survey meter TCS-171B (Hitachi Aloka Medical, Ltd., Miyagi, Japan). Measurements were made at μSv per hour, time-constant set to 10 s, and recorded when the readings were stabilized after a minimum of 30 s. The air dose rate was measured at the site where each trap was set, and the mean rate was calculated. In this study, ambient dose measurements (μSv) were treated as equivalent to external dose (μGy) in large Japanese mice. This decision was based on the small conversion factor for ambient dose rates to absorbed dose via specific air kerma, particularly for Cs isotopes [29].

### 2.4. Internal Exposure to Radiation

It is well known that radionuclides such as ^137^Cs and ^90^Sr become incorporated into teeth during their formation, accumulating within them until they naturally shed. The concentration of radioactivity in teeth is directly proportional to the amount of incorporated radionuclides within the body (see Supplemental Figures S1 and S2a,b). Therefore, teeth can serve as a reliable index for assessing internal exposure to radiation. In this study, we utilized imaging plates to measure the radioactivity of the growing incisor teeth of the large Japanese field mouse, expressing the results as quantum level (QL) values. This method allows for the estimation of individual internal radiation exposure. Further details regarding the methodology were described previously [30].

### 2.5. Sample Preparation for Light Microscopy (LM) and Transmission Electron Microscopy (TEM)

After collection, testicular samples, divided into 4–5 pieces from each group (control, low-, intermediate-, and high-level LDR radiation), were sectioned and immediately fixed in 2.5% glutaraldehyde (Sigma-Aldrich, St. Louis, MO, USA) in PBS, at 4 °C for at least 48 h before processing for LM and TEM as previously described [31,32]. Semithin sections (1 µm thick) were stained with methylene blue and images were taken using a digital camera (Leica DFC230) mounted on a LM (Zeiss Axioscope, Carl Zeiss Microscopy GmbH, Jena, Germany). Ultrathin sections (60–80 nm) were cut with a diamond knife, mounted on copper grids, and contrasted with UranyLess and Lead citrate (SIC, Rome, Italy). Images were captured using Philips TEM CM100 electron microscopes operating at 80 kV [33,34,35].

### 2.6. Morphometric Analysis

The diameter of 10 randomly selected seminiferous tubules per sample was assessed in each testis by drawing a straight line intersecting a transverse section of the tubule. To achieve this, ImageJ 1.53 software (http://rsbweb.nih.gov/ij/) (accessed on 30 January 2020) calibrated for 40× magnification was utilized, enabling the visualization and quantification of approximately five transverse sections of round-shaped tubules in each LM micrograph, as previously described [36]. The line was consistently applied to intersect the center of the tubule, and only round-shaped tubules were included in the analysis. Additionally, using the same software, the seminiferous epithelium height was measured in each testis. For this analysis, 10 transverse sections of round-shaped tubules per sample were examined in LM images captured at 40× magnification [36].

ImageJ 1.53 software (http://rsbweb.nih.gov/ij/) (accessed on 30 January 2020) was used to measure the mean intercellular space diameter and the numerical density of cytoplasmic vacuolization, vacuolated mitochondria, lipid droplets, acrosomal vesicles, and mitochondrial cristae on low-magnification TEM micrographs of control and LDR radiation groups [32,35]. More specifically, the numerical density of cytoplasmic vacuolization, vacuolated mitochondria, lipid droplets, acrosomal vesicles, and mitochondria cristae and the width of intercellular spaces were determined on at least five equatorial sections (distance between the sections: 3 μm) and was performed by analyzing TEM micrographs at 2600×. Values were expressed as micrometers (µm) for the mean intercellular space diameter between spermatogenic cells (at least 15/micrograph/group) of the basal layer or as numerical density per 500 μm^2^ of the tubule surface area.

### 2.7. Statistical Analysis

All data were expressed as means ± standard deviation (SD). Statistical comparisons were performed using one-way ANOVA with Tukey honest significant difference (HSD) tests for post hoc analysis (GraphPad InStat 10.1.2. GraphPad Software, La Jolla, San Diego, CA, USA). Differences in values were considered significant if *p* < 0.05.

## 3. Results

### 3.1. Control

**Figure 2:**
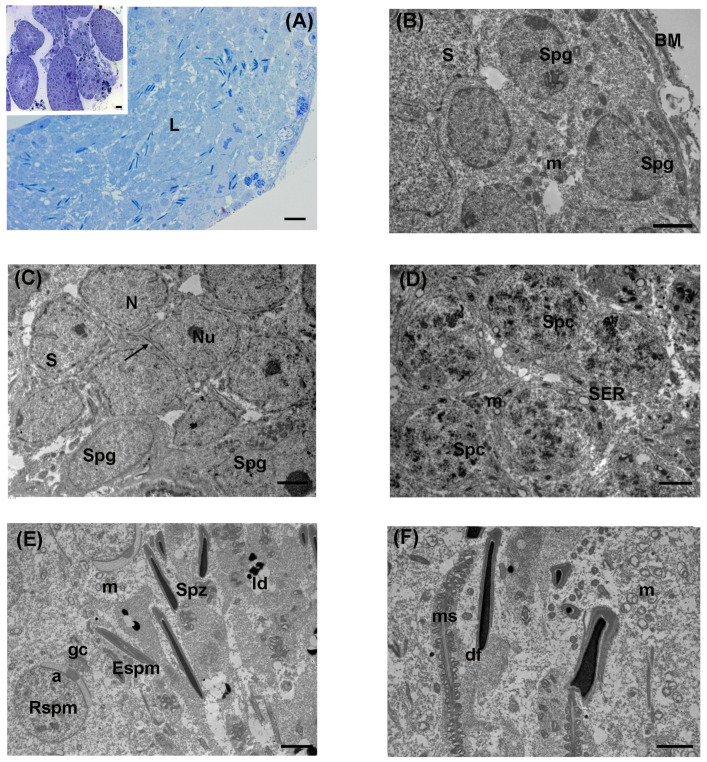
Ultrastructure of testis from the control group of the large Japanese field mouse. (**A**) Representative semithin section of spermatic tubules showing the germinative epithelium with cells at different stages of maturation. L: lumen; LM magnification: 40×; scale bar: 10 µm. Inset in (**A**) Representative image of seminiferous tubules at low magnification; LM magnification: 20×; scale bar: 10 µm. (**B**) Spermatogonia (Spg) were adherent to the basement membrane (BM) of spermatic tubules. The nucleus contained patches of heterochromatin. Numerous mitochondria (m), with evident cristae, were present in their cytoplasm. S: Sertoli cell; TEM scale bar: 2 µm. (**C**) TEM micrograph showing a group of Sertoli cells (S) with large, indented nucleus (N) and prominent nucleolus (Nu). A tight junction (arrow) appears between the cell membranes of adjacent Sertoli cells above the dark spermatogonia (Spg); TEM scale bar: 2 µm. (**D**) A group of primary spermatocytes (Spc) with normal nuclei were present toward the lumen. The cytoplasm contains multiple aggregated mitochondria (m) and numerous vesicles of smooth endoplasmic reticulum (SER); TEM scale bar: 2 µm. (**E**) The adluminal compartment showed round spermatids (Rspm) were at the cap-phase, with electron-dense pro-acrosomal vesicle (a) and the Golgi complex (gc). Toward the lumen, elongated spermatids (Espm) and longitudinal sections of spermatozoa (Spz) head were visible; m: mitochondria; ld: lipid droplets; TEM, scale bar: 2 µm. (**F**) TEM micrograph of a longitudinal section of midpiece from spermatozoa tails and heads; ms: mitochondrial sheath; df: dense fibers; m: mitochondria; TEM, scale bar: 2 µm.

In the control group, LM analysis on semithin sections showed well-preserved seminiferous tubules (Figure 2A). Tubules were delimited by regular basal membranes surrounded by myoid cells and filled by a stratified seminiferous epithelium, rich in differently staged germ cells. Spermatogonia mainly appeared as pale, big, and rounded cells laid in the outer basal compartment, with intensely stained ovoid nuclei and evident spots of heterochromatin. Primary spermatocytes, with rounded nuclei, were distributed in one or two rows. Numerous elongated spermatids, with intensely stained nuclei, and spermatozoa were present in the adluminal compartment. Somatic Sertoli cells, of prismatic shape, frequently extended from the basal to the adluminal compartment (Figure 2A).

TEM evaluation confirmed the normal ultrastructure of seminiferous tubules. Spermatogonia were in the basal compartment, adherent to the basal lamina; they appeared as large ovoid cells characterized by roundish nuclei with peripheral electron-dense patches of heterochromatin and scattered mitochondria (Figure 2B). Sertoli cells were identified by their large indented euchromatic nuclei and prominent nucleoli (Figure 2C). Their cytoplasm, often located from the basal to the adluminal compartment, was rich in electron-dense lipid droplets. The second layer of the basal compartment was represented by round primary spermatocytes, with large nuclei and patches of heterochromatin. Their cytoplasm presented a rich pool of mitochondria, often aggregated, with well-evident cristae (Figure 2D). Frequently, it was possible to identify the formation of the head cap at the cap phase. Numerous round spermatids were present at the cap phase, showing that the acrosomal granule and its membrane encircled the cell with the shape of a cap, or at the acrosomal phase, with condensed acrosome and evident Golgi apparatus (Figure 2E). Elongated spermatids were visible at the maturation phase, with cone-like electron-dense pro-acrosomal vesicles, towards the tubular lumen. 

Numerous immature spermatozoa were present throughout the lumen, together with transversal sections of the middle, principal, and end pieces (Figure 2E,F). Cross sections in the middle pieces of the spermatozoa showed a central axoneme, surrounded by nine outer dense fibers, the mitochondrial sheath, and the cell membrane (Figure 2F).

### 3.2. Low Levels of LDR Radiation (Tanashio Site)

**Figure 3:**
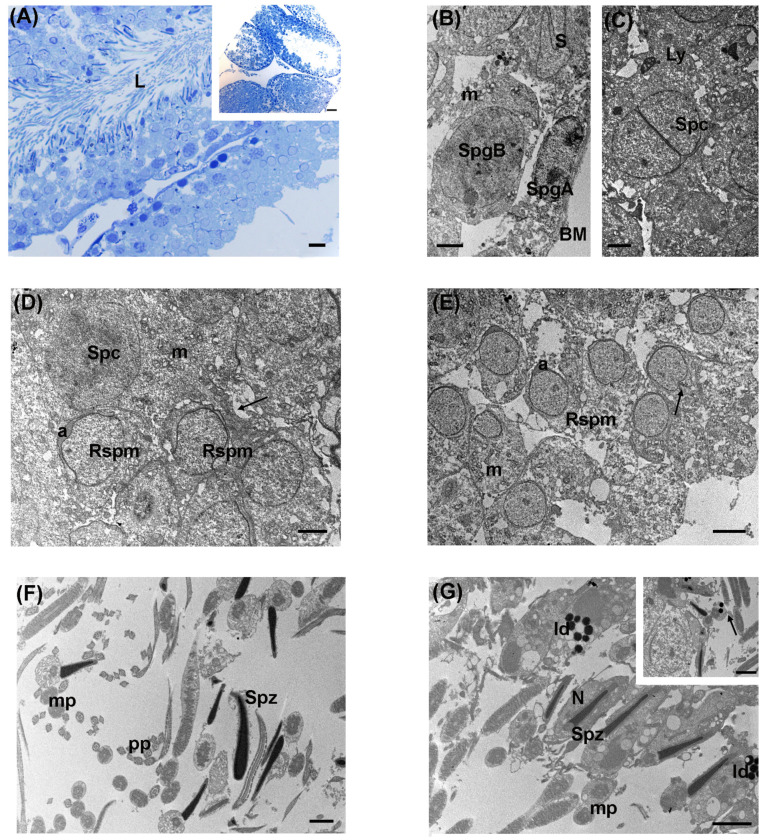
Ultrastructure of large Japanese field mouse testis exposed to low level of LDR radiation. (**A**) Representative semithin section of a spermatic tubule densely populated by the germinative epithelium in the lumen (L); LM magnification: 40×; scale bar: 10 µm. Inset in (**A**) Representative image of seminiferous tubules at low magnification; LM magnification: 20×; scale bar: 50 µm. (**B**) Basal compartment of a seminiferous tubule delimited by the basement membrane (BM), containing type A-pale spermatogonia (SpgA) with round nuclei and patches of heterochromatin, and type B-dark spermatogonia (SpgB) with evident nucleolus. Several mitochondria (m) were visible in the cytoplasm; S: Sertoli cell; TEM, scale bar: 2 µm. (**C**) TEM micrograph showing a primary spermatocyte (Spc) in the meiotic division; Ly: secondary lysosome; TEM, scale bar: 2 µm. (**D**) Representative TEM image showing the adluminal compartment containing spermatocyte (Spc), with patches of heterochromatin, several round spermatids (Rspm) at cap-phase, and the pro-acrosomal vesicle (a); note the wide intercellular space between spermatocytes and roundish spermatids (arrow); m: mitochondria; TEM, scale bar: 2 µm. (**E**) TEM micrograph showing a portion of the adluminal compartment with numerous roundish spermatids (Rspm) during the elongation process and the formation of acrosome (a) together with the primitive centriole (arrow); m: mitochondria; TEM, scale bar: 5 µm. (**F**) The adluminal compartment showed numerous immature spermatozoa (Spz) with cross-sections of midpiece (mp) and principal piece (pp) from the tails and heads; TEM, scale bar: 2 µm. (**G**): Low magnification of the lumen occupied by numerous heads and tails of immature spermatozoa (Spz) with evident clusters of electron-dense lipid droplets (ld); mp: midpiece, N: nucleus; TEM, bar: 2 µm. Inset in (**G**) lipid droplets (arrow) were visible in the cross-section of spermatozoa tail; TEM, scale bar: 2 µm.

Following low levels of LDR exposure in *A. speciosus*, LM evaluation of testes showed a general good preservation of seminiferous tubules, delimited by a thick and continuous basal lamina, with outer myoid cells. The lumen was narrow in most spermatic tubules and densely filled with the germinal epithelium (Figure 3A). It was possible to distinguish germinal cells at different stages of spermatogenesis. Type A spermatogonia were in the basal position, possessing pale ovoid nuclei with intensely stained chromatin. Occasionally, type B spermatogonia, with rounder nuclei and heterochromatin, were also seen near the basal lamina. More distant to the basal lamina, numerous spermatocytes appeared at the preleptotene and leptotene stages. Spermatocytes at the diplotene and pachytene stages were observed in the inner layer. Numerous early-round spermatids were recognized by the proacrosomal vesicle. Elongated spermatids were dispersed among Sertoli cells and identified by nuclei with intensely stained chromatin (Figure 3A).

By TEM analysis, type A dark and pale spermatogonia were present at the basal membrane level and Sertoli cells were recognizable by their indented nuclei (Figure 3B). Towards the tubular lumen, groups of primary spermatocytes, with large and rounded nuclei, showed patches of heterochromatin. In their cytoplasm, a rich pool of well-preserved mitochondria, with evident cristae, was distinguishable. Occasionally, spermatocyte meiosis was recognizable and secondary lysosomes were sometimes detected in their cytoplasm (Figure 3C,D). Wide intercellular spaces were present between spermatocytes (Figure 3D). Notably, cytoplasmic holes and vacuoles were occasionally found between spermatogonia, spermatocytes, and spermatids (Figure 3B–D). In the second layer of the germinal epithelium, round spermatids, with the formation of the head cap-like structure, were observed at the cap phase. Most of them showed a proacrosomal vesicle, delimited by a plasma membrane (Figure 3D,E). Elongated spermatid nuclei at the maturation phase protruded in the adluminal compartment, characterized by electron-dense acrosomal vesicles, at the acrosomal phase. Immature spermatozoa were found in the center of the lumen, together with numerous cross-sections of the principal piece, middle piece, and end piece (Figure 3F,G). Notably, numerous electron-dense lipid droplets were visible in the spermatogenic cells (Figure 3G). Differently from controls, they were occasionally found in the sperm midpiece (inset, Figure 3G). Interstitial cells appeared electron pale.

### 3.3. Intermediate Levels of LDR Radiation (Ide Site)

**Figure 4:**
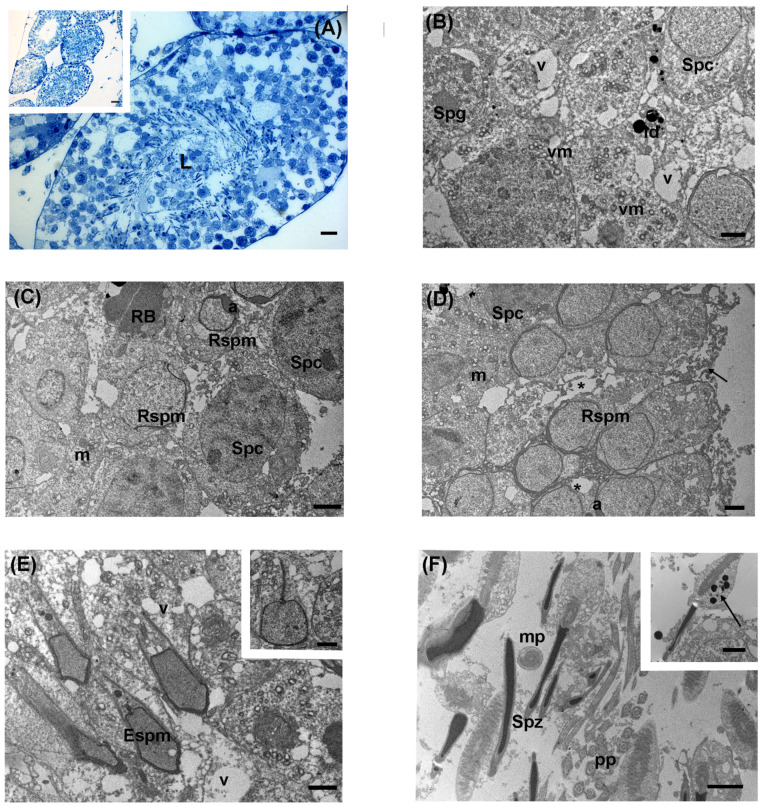
Ultrastructure of large Japanese field mouse testis from the intermediate levels of LDR radiation. (**A**) Representative semithin section of seminiferous tubules densely filled with the germinal epithelium; L: lumen; LM magnification: 40×; scale bar: 10 µm. Inset in (**A**) Representative image of seminiferous tubules at low magnification; LM magnification: 20×; scale bar: 10 µm. (**B**) In the tubular compartment, spermatogonia (Spg), with heterochromatin flakes, were adherent at the basal membrane level. Primary spermatocyte (Spc) with a rounded large nucleus. Vacuolated mitochondria (vm) in a spermatocyte cytoplasm. v: vacuoles; ld: lipid droplets; TEM, scale bar: 2 µm. (**C**) Spermatocytes (Spc) constituted the second line of the germinal epithelium; round spermatids (Rspm) at the cap-phase or acrosomal phase were visible, together with residual bodies (RB); a: pro-acrosomal vesicle; m: mitochondria; TEM, scale bar: 2 µm. (**D**) TEM micrograph displayed several round spermatids (Rspm) at cap-phase or pro-acrosomal phase with electron-dense pro-acrosomal vesicle (a). Note the intercellular wide space between the cells (*). Cell fragments were visible (arrow); m: mitochondria; Spc: spermatocytes; TEM, scale bar: 2 µm. (**E**) Numerous elongated spermatids (Espm), with cone-like electron-dense pro-acrosomal vesicles, protruded in the lumen of the seminiferous tubule. Diffuse vacuolization was visible; v: vacuoles; TEM, scale bar: 2 µm. Inset in (**E**) Detail of round spermatid at the cap-phase: the centriole has contacted the nucleus, and the tail has started to form; TEM, scale bar: 2 µm. (**F**) TEM micrograph showing lumen compartment in which numerous heads of immature spermatozoa (Spz) were visible, along with cross and longitudinal sections of the midpiece (mp) of tails; pp: principal piece; TEM, scale bar: 2 µm. Inset in (**F**) Spermatozoa with lipid droplets (arrow) in the midpiece; TEM, scale bar: 2 µm.

Upon exposure to intermediate levels of LDR radiation, through LM analysis, the organization of seminiferous tubules showed overall good preservation with a continuous basal lamina, as in the previous groups of control and low-level LDR exposure. Numerous cellular elements were present in the lumen (Figure 4A). Sertoli cells were adherent to the basal lamina. Adjacent to Sertoli cells, type A spermatogonia were present, characterized by ovoid nuclei with evident heterochromatin. Moving from the outer layer toward the lumen of the seminiferous epithelium, numerous spermatocytes, at different phases of spermatogenesis, were found. Particularly, it was possible to recognize several cellular elements at the leptotene and diplotene stages. More luminally, spermatocytes at pachytene stages were distinguished. Compared to the previous group, round spermatids and acrosomal vesicles were less abundant. Inside the lumen, numerous elongated spermatids were observed. Among them, numerous residual bodies of late spermatids were present (Figure 4A).

Via TEM, spermatogonia showed a central nucleus with evident patches of chromatin and a rich pool of mitochondria in the cytoplasm (Figure 4B). Throughout the germinal epithelium, spermatocytes constituted the second line of the seminiferous tubule. Lipid droplets were dispersed in the germinal epithelium (Figure 4B). The proacrosomal vesicle was visible in roundish spermatocytes. In addition, wide intercellular spaces were recognizable among spermatocytes (Figure 4C). Round spermatids were observed at the cap phase, where the acrosomal granule and its membrane surrounded the cell; at the acrosomal phase, a condensed acrosome was easily recognizable (Figure 4C,D). In the adluminal compartment, it was possible to recognize elongating spermatids with growing flagella (Figure 4E). In contrast to the other groups, an increase in vacuolization was observed, and the mitochondria, which were mostly aggregated, sometimes exhibited less visible cristae (Figure 4E).

Testicular immature spermatozoa were present in the adluminal compartment. Ultrastructural analysis evidenced distinguishable sperm heads, with electron-dense nuclei, and the flagellum organized in midpiece (with the mitochondrial sheath regularly arranged), principal piece, and end piece, as also shown by the numerous transversal sections of the tail (Figure 4F). Rarely, the midpiece of sperm presented electron-dense lipid droplets (inset, Figure 4F).

### 3.4. High Levels of LDR Radiation (Omaru Site)

**Figure 5:**
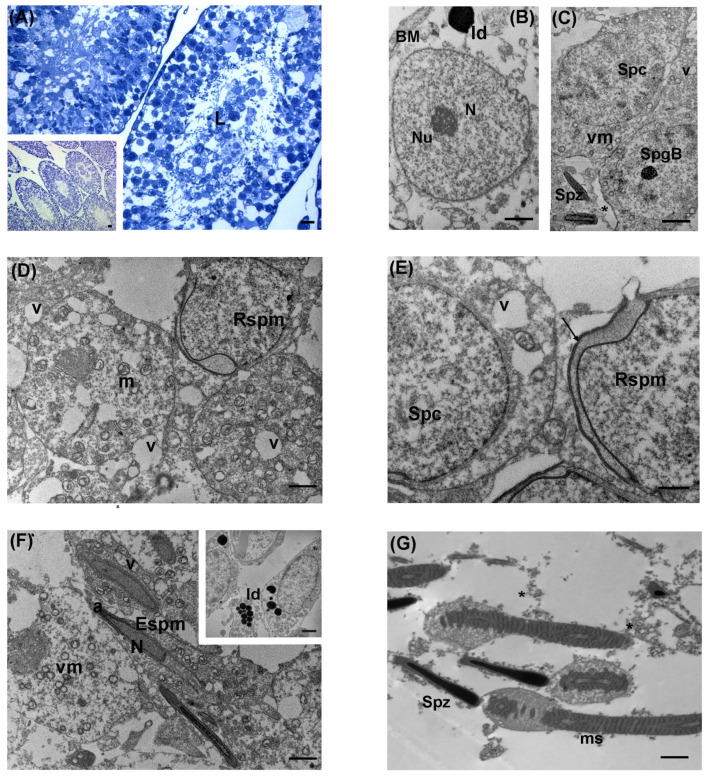
Ultrastructure of large Japanese field mouse testis exposed to high levels of LDR radiation. (**A**) Representative semithin section of a spermatic tubule densely populated by the germinative epithelium in the lumen (L); LM magnification: 40×, scale bar: 10 µm. Inset in (**A**) Representative image of seminiferous tubules at low magnification; LM magnification: 10×; scale bar: 2 µm. (**B**) In the basal compartment, a Sertoli cell with regular indented nucleus (N) and evident nucleolus (Nu) adherent at the basal membrane (BM); ld: lipid droplets; TEM, scale bar: 2 µm. (**C**) TEM image of type B spermatogonia (SpgB) and spermatocyte (Spc). Note the wide intercellular space between the cells (*); vm: vacuolated mitochondria, v: vacuoles; Spz: spermatozoa; TEM, scale bar: 2 µm. (**D**) In the adluminal compartment, round spermatids (Rspm) at the cap-phase were visible. Numerous scattered mitochondria (m) with evident cristae were found; v: vacuoles; TEM, scale bar: 2 µm. (**E**) High magnification of spermatocytes (Spc) with vacuoles in their cytoplasm and round spermatids (Rspm) with cap-like structure and its membrane (arrow); v: vacuoles; TEM, scale bar: 1 µm. (**F**) Elongated spermatids (Espm) during the elongating process with cone-like electron-dense pro-acrosomal vesicle (a) and condensed nuclei (N). In their cytoplasm, vacuolated mitochondria (vm) were also visible; v: vacuoles; TEM, scale bar: 2 µm. Inset in (**F**) a detail of numerous lipid droplets (ld) present in the spermatogenic cells; TEM, scale bar: 2 µm. (**G**) Immature spermatozoa (Spz) protruded in the lumen with a longitudinal section of the midpiece and head. Cell fragments were visible (*). ms: mitochondrial sheath; TEM, scale bar: 1 µm.

After high levels of LDR exposure, LM revealed overall good preservation of seminiferous tubules. The lumen appeared populated by numerous cellular elements (Figure 5A). Sertoli cells were adherent to the basal lamina. Numerous type A spermatogonia were present in the basal compartment. Throughout the seminiferous tubules, it was possible to recognize different phases of spermatogenesis. Numerous spermatocytes, at the leptotene and diplotene stages, were found. Moving from the basal to the adluminal compartment, spermatocytes at the pachytene stage were distinguished. The second layer was characterized by round spermatocytes, with an initial pattern of chromatin condensation. Round spermatids and their acrosomal vesicles were less present in the observed sections, whereas inside the lumen, elongated spermatids with acrosomal membranes were visible. Sometimes, residual bodies were present mostly in the adluminal compartment (Figure 5A).

By TEM, the testis ultrastructure was generally similar to the previous groups, except for some characteristics. Spermatogonia and Sertoli cells were adjacent to the basal lamina (Figure 5B). Towards the tubular lumen, groups of spheric spermatocytes showed scattered mitochondria, often aggregated and with a round-to-ovoid shape (Figure 5C). However, sometimes mitochondrial cristae appeared less electron-dense or vacuolated in the spermatocytes (Figure 5C–F). Wide intercellular spaces were visible between spermatogenic cells (Figure 5C). The formation of a head cap-like structure at the cap phase with the acrosomal membrane was well visible (Figure 5D,E). Roundish spermatids were present at the acrosomal phase (Figure 5D,E). Elongated spermatids, at the maturation phase, were interspersed in the lumen with numerous immature spermatozoa and their cross sections (Figure 5F,G). Vacuolated cells were found (Figure 5G). Leydig cells were seen in the interstitial tissue; they showed a large and slightly rounded nucleus with peripheral heterochromatin and a prominent nucleolus. Similarly, to the other exposed groups, numerous electron-dense lipid droplets were visible in the spermatogenic cells (inset, Figure 5F). Occasionally, some cell fragments were present, and cytoplasmatic vacuolization was found (Figure 5F,G).

### 3.5. Morphometric Analysis

Morphometric analysis on the seminiferous tubules diameter and seminiferous epithelium height did not show statistical differences among controls and exposed groups (*p* > 0.05; Table 2). Differently, the mean intercellular space showed an increasing trend after ionizing radiation exposure between controls (0.88 ± 0.27) and experimental groups (1.10 ± 0.3, 1.17 ± 0.32 and 1.7 ± 0.40, low, intermediate, and high levels of LDR radiation, respectively). The increase was highly significant between controls/low levels and high levels of LDR radiation groups (Table 3, *p* < 0.001). Cytoplasmic vacuolization also significantly increased in exposed groups when compared to controls (5.6 ± 2.3 vs. 11.6 ± 1.5, 14.8 ± 2.0 and 14.2 ± 3.5 respectively; *p* < 0.05) (Table 3).

The morphometric evaluation of vacuolated mitochondria showed an upward trend in exposed samples (5.4 ± 1.6 vs. 11.6 ± 3.4 and 13.2 ± 2.5, respectively; *p* > 0.05) (Table 3), even if there was no statistical significance among groups.

The morphometric analysis on the TEM micrograph evidenced a rising trend in the numerical density of lipid droplets from control to all levels of LDR radiation groups even if not significant (controls: 5 ± 2.5; low levels of LDR radiation: 6.8 ± 5.3; intermediate levels of LDR radiation: 7.2 ± 2.3; high levels of LDR radiation: 12 ± 4.2; *p* > 0.05). However, a significant increase was found at the high levels of LDR groups, compared to controls (5 ± 2.5 vs. 12 ± 4.2; *p* < 0.05) (Table 3).

Morphometry about the numerical density of acrosomal vesicles showed a reduction from controls to highly exposed groups, with a significant decrease in the intermediate group (2 ± 1 vs. 4.6 ± 2.0, intermediate levels of LDR radiation vs. control group, respectively; *p* < 0.05). Morphometric assessment of the numerical density in electron-dense mitochondrial with no visible cristae indicated an upward trend, with significant differences in controls compared to the intermediate- and high-level LDR radiation groups (5.2 ± 0.8 vs. 8.8 ± 2.4 and 9.2 ± 1.9, respectively; *p* < 0.05) (Table 3).

## 4. Discussion

The radiation impacts on wildlife in the FDNPP-contaminated areas have been studied in animals, insects, and plants, and present data showed either significant or non-significant radiation effects [29,36]. Radiation exposure also affects reproductive functions, particularly in the testis, as it is one of the most radiosensitive organs [37]. Here, we investigated the testicular ultrastructure of *A. speciosus*, albeit on a low number of samples, aiming to gain insights into the potential reproductive impacts associated with various radiation exposure dose rates caused by the release of radioactive material from the FDNPP accident. We collected wild mice from three contaminated areas within the ex-evacuation zone, and we considered their chronic external exposure as low (0.29 μGy/h), intermediate (5.11 μGy/h), and high (11.80 μGy/h) LDR radiation for our analysis. 

*A. speciosus*, being a seasonal breeder, undergoes specific seasonal variations in the morphology of its seminiferous tubules. This seasonal breeding pattern involves regulated testis activation and involution. During the seasonal reproductive cycle, the regulation of spermatogenesis is controlled by proliferation and apoptosis in male germ cells, including spermatogonia and primary spermatocytes [12,21]. Furthermore, previous studies reported differences in radiation sensitivity between spermatogonia and spermatocytes [38], even among the various stages of the spermatogenic cycle [37,39,40]. As spermatogenesis serves as an indicator of ecological factors influencing animal reproduction, it holds significance in the context of studying radiation effects on reproductive systems.

In our study, the ultrastructural analysis revealed generally well-preserved seminiferous tubules in all groups studied, with occasional signs of changes. TEM observations displayed no remarkable modifications in the ultrastructure of spermatogonia, spermatocytes, spermatids, and elongated spermatids, suggesting a normal process of spermatogenesis in all LDR radiation groups compared with controls. Seminiferous tubules diameter and germinal epithelium height did not change among experimental groups. Small differences evidenced by our data involve the presence of wide intercellular spaces between spermatogenic cells, which may be associated with oxidative stress [41], fluid accumulation, and tubular edema [42,43,44]. Morphometric analysis evidenced that the increase in the intercellular space was highly significant in control and high-levels-of-LDR-radiation groups, with significant differences also being revealed among exposed groups, further conforming ultrastructural data.

The cytoplasmic vacuolization, mostly observed in the groups exposed to intermediate and high levels of LDR radiation, was confirmed to significantly increase in the morphometric analyses in all exposed groups compared to controls. Vacuolization may result from the dilatation and vesiculation of the endoplasmic reticulum due to phagocytic vacuoles remaining after the digestion of apoptotic germ cells [41,45]. These findings could indicate physiological dynamics in murine spermatogenesis during the seasonal reproductive cycle. Indeed, in seasonal reproductive animals such as *A. speciosus*, the spermatogenic process is divided into three phases—inactive, transient, and active—which are regulated by proliferation and apoptosis [12,21]. These mechanisms control the development and number of germ cells in the testis and undergo variations at different points during the phases of the spermatogenic cycle. As shown previously, the proliferation capacity of germ cells increases during the transitional and active phases and decreases in the inactive phase. On the other hand, spontaneous apoptosis increases during the inactive phase and decreases during the active and transitional phases [21]. In contrast, Takino and colleagues [19] (2017) reported a lower frequency of apoptosis in the testis germ cells of *A. speciosus* inhabiting the Fukushima ex-evacuation zone and exposed to external dose rates between about 1 and 18 μGy/h. While these findings could potentially be attributed to LDR radiation exposure, it is worth noting that the animals were captured during the breeding season, i.e., at the end of their active phase [12,21]. The presence of cytoplasmic vacuolization can be indicative of increased apoptosis, marking the onset of the transitional phase, which is a normal part of the spermatogenesis process in large Japanese field mice.

Moreover, the cell fragments indicated by our results support the hypothesis of apoptosis, as previously demonstrated by other authors [46,47]. In the testis, the physiological apoptotic death of selected germ cells plays an important role in limiting the germ cell population [48,49,50]. Thus, it can be assumed that the morphological data related to apoptosis are dependent on the normal process of spermatogenesis. It remains to be clarified whether prolonged exposure to ionizing radiation may have interfered with apoptosis.

Our results also showed the presence of vacuolated mitochondria, mostly in the high levels of LDR groups. The morphometric analysis revealed, however, that the increase in mitochondrial vacuolization was significant when exposed groups were compared to controls. Furthermore, the numerical density of electron-dense mitochondria with no evident cristae increased in exposed groups. Ultrastructural and morphometric data confirm what was previously proposed by others, namely, that mitochondria with this morphology may be due to the excessive production of reactive oxygen species (ROS) and could be indicative of mitochondrial stress [36,41,46]. Mitochondria are highly dynamic organelles [51,52], undergoing significant changes in morphology, size, and localization during spermatogenesis, as reported by several studies. Additionally, mitochondrial dynamics in spermatogenesis demonstrate developmental stage-specific regulation, with morphological alternations that prepare their structural adaptation to fit within the sperm flagellum [53].

After exposure to LDR radiation, TEM analysis revealed a significant increase in the numerical density of lipid droplets in the spermatogenic cells, between mice exposed to high levels of LDR groups and controls. Interestingly, increased lipid droplets were commonly found in radioresistant cancer cells as a stress response. Their presence was explained by a decrease in lipid peroxidation induced by ionizing radiation and accounts for an improved antioxidant ability due to higher ROS levels [54,55,56]. Data from previous studies have shown that stem spermatogonia were not killed at low-dose irradiation in mice and immediately began repopulating the seminiferous epithelium with different sperm cells [57]. Recent research has suggested that the recovery of spermatogenesis in rats can be improved by administering steroid hormones before irradiation exposure. These hormones work by reducing luteinizing hormone levels, subsequently decreasing testosterone production, and facilitating the completion of spermatogenesis [58,59,60]. Furthermore, Leydig cells show high resistance to radiation, potentially due to an observed increase in antioxidants after irradiation. However, the precise molecular mechanisms driving these cellular responses remain largely unknown, although physiological and cellular responses to ionizing radiation in the testes are well documented. Intriguingly, studies have revealed that supplementing exogenous testosterone inhibits the GnRH-antagonist-stimulated recovery of spermatogenesis in irradiated rats, contrary to the hormone’s typical actions [60,61,62]. Considering these concerns, our findings suggest that Leydig cells exert a protective effect on sperm cells exposed to ionizing radiation. 

Since our ultrastructural results may be attributable to changes related to the cell turnover of spermatogenesis, it cannot be excluded that changes found in the morphology of the testis of *A. speciosus* are due to physiological dynamics during the usual mechanism of spermatogenesis.

Data obtained in this work are in agreement with previous studies demonstrating no significant differences in the male reproductive system of wild animals exposed to LDR radiation after the Fukushima accident [19,26,27].

The observed absence of significant differences between the morphology of unexposed testes and those exposed to a dose rate of less than 100 μGy/h aligns with the threshold suggested by UNSCEAR (2008), indicating that effects on population integrity in most terrestrial communities are unlikely to occur below this dose rate [3]. The highest level of LDR radiation in this study had an ambient dose rate of 11.8 μSv/h at a measurement height of 1 m. However, if measured at ground level or underground, where the mice spend much of their time, the dose rates would be higher [63]. 

The QL values of teeth in each animal (see Supplemental Figure S1) ranged from 1018 to 8663, and this is directly proportionate to the body burden of each animal or the activity concentration of radioactive substances, denoted as Bq/kg. This means that the internal doses would not cause the total dose rate to exceed 100 μGy/h, which may explain the lack of discernible changes in the testicular ultrastructure of *A. speciosus*.

Given that the mouse testis shows sensitivity to low-dose irradiation similar to that observed in humans [57], these findings may provide valuable insights into the effects of ionizing radiation exposure on fertility preservation in humans. To support the establishment of dose limits, dose coefficients, and other radiological protection quantities applicable to humans, further studies are necessary to provide a more accurate estimate of total radiation exposure.

## 5. Conclusions

In conclusion, although some modifications were present in the testes ultrastructure, the levels of chronic LDR radiation exposure observed in this study, associated with the FDNPP, did not have an evident impact on spermatogenesis in large Japanese field mice.

Responses to radiation exposure can be various and may depend on the type of source, effective dosages of irradiation, duration of exposure, and mostly on individual genetic assessment and spermatogenesis physiology.

In this study, the ultrastructural and morphometric differences observed in the seminiferous tubules of *A. speciosus* suggested the involvement of the active phase of spermatogenesis in restoration and protection from the detrimental effects exerted by ionizing radiation thanks to cell proliferation, apoptosis, and the increased production of lipid droplets as stress responses due to the antioxidant activities. It is likely that the physiological mechanisms of male germ cell proliferation and apoptosis, commonly observed in seasonally breeding animals, aid in abnormal cell removal, thus contributing to the normal progression of spermatogenesis. These data improve our knowledge of the effect of long-term exposure to LDR radiation on the male reproductive system of wild animals and may be used as the morphological basis for further studies assessing the male reproductive potential of wild animals. Our findings also highlight the importance of evaluating the spermatogenetic process in seasonal breeders as an indicator of ecological factors. However, the available data are still limited due to the uniqueness of the samples, the complexity of spermatogenesis mechanisms, and environmental factors such as the breeding season. Consequently, an accurate prediction of the detrimental effects connected to LDR radiation exposure on the male reproductive system, particularly in the context of the Fukushima accident, remains challenging. Further investigation about the effect of ionizing radiation on spermatogenesis should be extended to a higher number of animals in the ex-evacuation zone of the FDNPP, if available, to confirm the absence of radiation effects.

## Figures and Tables

**Table 1:** Individual data for each large Japanese field mouse.

Group	Number of Animals	ID	Site	Breeding Season	Sampling Date	Body Weight (g)	Ambient Dose Rate (µSv/h)	Average Diameter of Seminiferous Tubule (µm)
**Niigata** **(Control)**	4	Ni_36	Kakuta	TP	02/06/2021	61.6	0.048 *	200.3
		Ni_38	Kakuta	TP	02/06/2021	52.1	0.049 *	144.8
		Ni_41	Kakuta	TP	02/07/2021	62.6	0.049 *	129.4
		Ni_44	Kakuta	TP	02/07/2021	45.7	0.048 *	138.7
**Fukushima**	4	Fu_730	Tanashio	RS	17/04/2018	39.3	0.29	244.7
		Fu_733	Omaru	RS	18/04/2018	39.8	11.80	245.5
		Fu_735	Ide	RS	18/04/2018	17.0	5.11	236.4
		Fu_737	Omaru	RS	19/04/2018	28.8	11.80	243.7

* Data obtained from monitoring post (15 km from Kakuta site; data was retrieved from https://radioactivity.nra.go.jp/ja/list/603/list-1.html on 10 October 2023). TP: Transitional phase; RS: Reproductive season.

**Table 2:** Seminiferous tubules diameter and germinal epithelium height (expressed as mean ± standard deviation) in control and LDR radiation (low-, intermediate-, high-level) groups. Morphometry was performed on LM images using one-way ANOVA with Tukey HSD post hoc analysis. Different superscripts indicate a significant difference (*p* < 0.05).

	Controls (Niigata Site)	Low Levels of LDR Radiation (Tanashio Site)	Intermediate Levels of LDR Radiation (Ide Site)	High Levels of LDR Radiation (Omaru Site)
**Seminiferous tubules** **Diameter (µm)**	172.5 ± 50.3 ^a^	244.7 ± 75.8 ^a^	236.4 ± 63.9 ^a^	245.8 ± 67.4 ^a^
**Germinal epithelium** **Height (µm)**	56.0 ± 15.5 ^a^	47.8 ± 10.1 ^a^	47.6 ± 11.8 ^a^	52.8 ± 16.4 ^a^

**Table 3:** Morphometric evaluation for TEM (expressed as mean ± standard deviation) of intercellular space, vacuolated mitochondria, cytoplasmic vacuolization, lipid droplets, acrosomal vesicles, and electron-dense mitochondria in controls and LDR radiation groups (low-, intermediate-, high-, level). Morphometry was performed using one-way ANOVA with Tukey HSD post hoc analysis. Different superscripts indicate a significant difference (*p* < 0.05).

	Controls (Niigata Site)	Low Levels of LDR Radiation (Tanashio Site)	Intermediate Levels of LDR Radiation (Ide Site)	High Levels of LDR Radiation (Omaru Site)
**Intercellular space (µm)**	0.88 ± 0.27 ^a^	1.10 ± 0.30 ^a,b^	1.17 ± 0.32 ^a,b^	1.7 ± 0.40 ^c^
**Cytoplasmatic vacuolization (500 µm^2^)**	5.6 ± 2.3 ^a^	11.6 ± 1.5 ^b^	14.8 ± 2.0 ^b^	14.2 ± 3.5 ^b^
**Vacuolated mitochondria (500 µm^2^)**	5.4 ± 1.6 ^a^	9.2 ± 2.5 ^a,b,c^	11.6 ± 3.4 ^b,c^	13.2 ± 2.5 ^b,c^
**Lipid droplets (500 µm^2^)**	5 ± 2.5 ^a^	6.8 ± 5.3 ^a,b^	7.2 ± 2.3 ^a,b^	12 ± 4.2 ^b^
**Acrosomal vesicles (500 µm^2^)**	4.6 ± 2 ^a^	2.6 ± 1.3 ^a,b^	2 ± 1 ^b^	2.4 ± 1.1 ^a,b^
**Electron-dense mitochondria (with no visible cristae) (500 µm^2^)**	5.2 ± 0.8 ^a^	6.8 ± 1.9 ^a,b^	8.8 ± 2.4 ^b^	9.2 ± 1.9 ^b^

## Data Availability

The data presented in this study are available on request from the corresponding author. The data are not publicly available due to privacy/ethical restrictions.

## Supplementary Materials

The following supporting information can be downloaded at https://www.mdpi.com/article/10.3390/biology13040239/s1: Figure S1: QL value for teeth incorporated in the whole body; Figure S2a: Correlation between the QL value of teeth and muscle; Figure S2b: Correlation between the QL value of teeth and liver.
