# Supplementary figures and images for: Ultrastructural Analysis of Large Japanese Field Mouse (Apodemus speciosus) Testes Exposed to Low-Dose-Rate (LDR) Radiation after the Fukushima Nuclear Power Plant Accident
Source: Biology (Basel). 2024 Apr 4;13(4):239. doi: 10.3390/biology13040239 (PMC11048324; doi:10.3390/biology13040239)

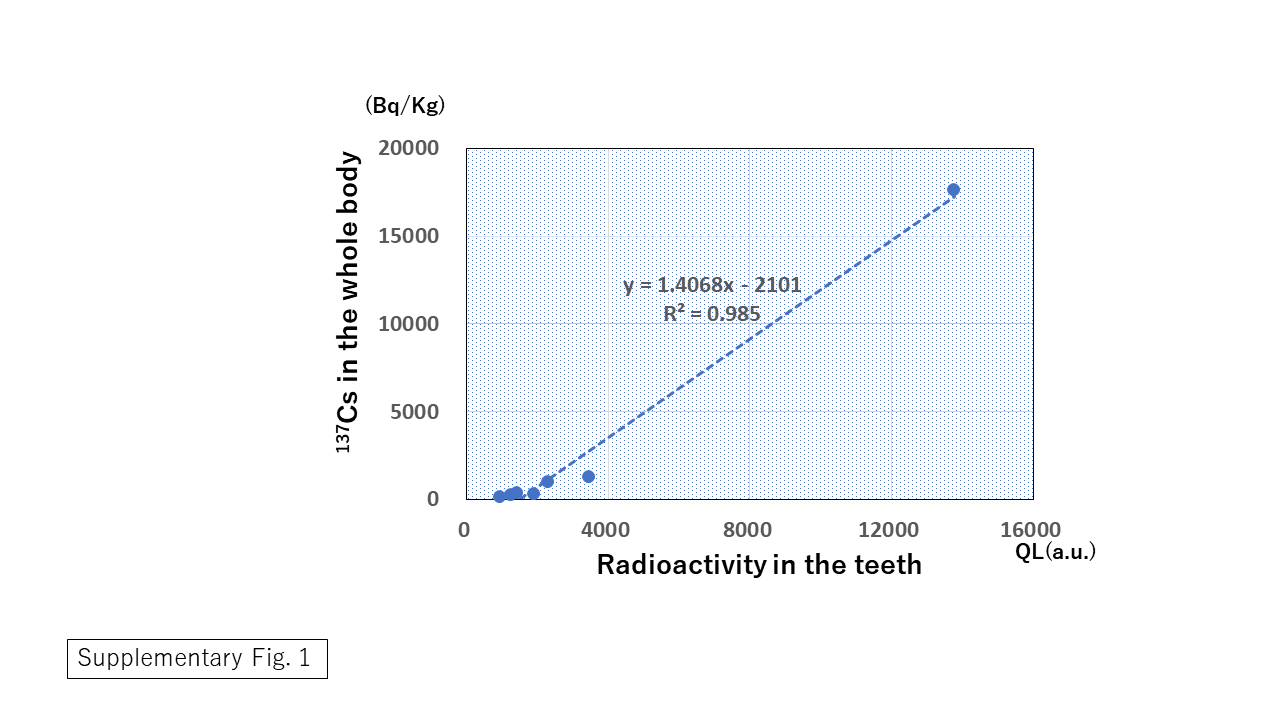

Supplement: Supplementary file 1 [file biology-13-00239-s001.zip › Supplementary/Supplementary Figure 1.tif]

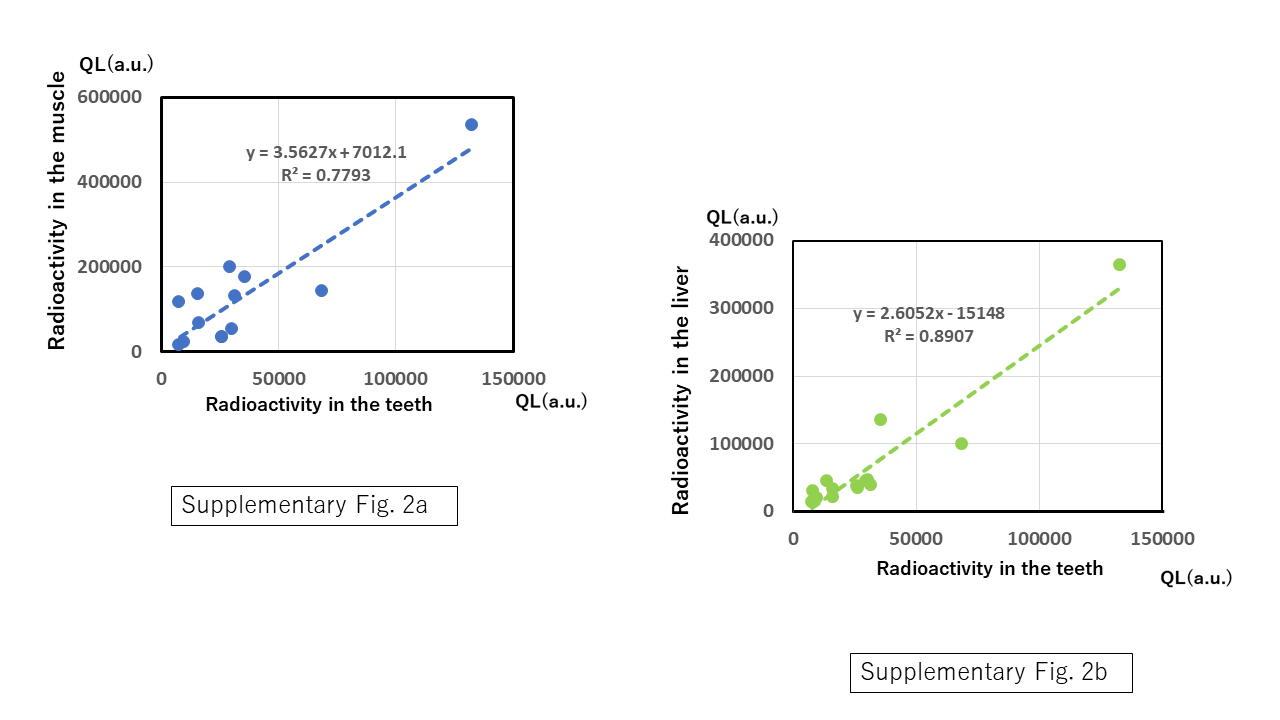

Supplement: Supplementary file 1 [file biology-13-00239-s001.zip › Supplementary/Supplementary Figure 2.tif]
